# Lentiviral Vector Mediated Thymidine Kinase Expression in Pluripotent Stem Cells Enables Removal of Tumorigenic Cells

**DOI:** 10.1371/journal.pone.0070543

**Published:** 2013-07-30

**Authors:** Tiong-Ti Lim, Caroline Geisen, Michael Hesse, Bernd K. Fleischmann, Katrin Zimmermann, Alexander Pfeifer

**Affiliations:** 1 Institute of Pharmacology and Toxicology, University of Bonn, Bonn, Germany; 2 NRW International Graduate Research School Biotech, PHARMA, Bonn, Germany; 3 Institute of Physiology I, University of Bonn, Bonn, Germany; 4 PharmaCenter Bonn, University of Bonn, Bonn, Germany; Baylor College of Medicine, United States of America

## Abstract

Embryonic stem cells (ES) and induced pluripotent stem (iPS) cells represent promising tools for cell-based therapies and regenerative medicine. Nevertheless, implantation of ES cell derived differentiated cells holds the risk of teratoma formation due to residual undifferentiated cells. In order to tackle this problem, we used pluripotent stem cells consisting of ES and iPS cells of mouse genetically modified by lentiviral vectors (LVs) carrying herpes simplex virus thymidine kinase (HSV-TK) under the control of different promoters of pluripotency genes. Cells expressing TK in turn are eliminated upon administration of the prodrug ganciclovir (GCV). Our aim was to study the conditions required for a safe mechanism to clear residual undifferentiated cells but using low MOIs of lentiviruses to reduce the risk of insertional mutagenesis. Our *in vitro* data demonstrated that TK expression in pluripotent stem cells upon treatment with GCV led to elimination of undifferentiated cells. However, introduction of hygromycin resistance in the LV transduced ES cells followed by pre-selection with hygromycin and GCV treatment was required to abolish undifferentiated cells. Most importantly, transplantation of pre-selected ES cells that had been transduced with low MOI LV in mice resulted in no teratoma development after GCV treatment *in vivo*. Taken together, our data show that pre-selection of ES cells prior to *in vivo* application is necessary if vector integration events are minimized. The study presented here gives rise to safer use of pluripotent stem cells as promising cell sources in regenerative medicine in the future.

## Introduction

Embryonic stem (ES) cells are derived from the primitive ectoderm of the inner cell mass of blastocysts [1,2]. They are characterized by their self-renewal capability and their pluripotency, i.e. they can develop into the three primary germ layers (ectoderm, endoderm, mesoderm) [3]. Because of their capacity to differentiate into all cell types of the adult body, ES cells became a promising source for cell-based therapies for regenerative medicine over the past years. However, the application of differentiated pluri- [4] or multipotent stem cells [5] for these approaches carries a potential risk of tumor (teratoma) formation *in vivo* due to residual undifferentiated cells in the transplanted cell population. Hence, removal of residual undifferentiated stem cells from the differentiated cell population has been considered as an essential requirement for use of stem cell-based therapies. In the light of ethical controversies around the usage of human ES cells, a number of groups demonstrated successful generation of induced pluripotent stem (iPS) cells from adult somatic cells [6–8]. Thus, iPS cells might also be used as an alternative source for stem cells in regenerative medicine or cell replacement therapies [8]. Also for these cells safety concerns about their tumorigenic potential have to be addressed.

Previous reports have proposed elimination of the undifferentiated cells using suicide genes [9–11]. The transfer of a suicide gene has even been successfully used in clinical trials for tumor elimination [12]. One of the most thoroughly studied and widely used approach is based on the herpes simplex virus thymidine kinase (HSV-TK) that converts the prodrug ganciclovir (GCV) to a toxic metabolite [12]. Various routes to deliver the transgene, including transfection or viral transduction, have been studied [10,11]. Moreover, approaches using cytotoxic antibodies against undifferentiated ES cells [13,14] or an antibody against a surface antigen of ES cells combining flow cytometry-based separation were used to remove undifferentiated pluripotent cells [15] before cell transplantations.

Lentiviruses are members of the *Retroviridae* family, which can stably integrate their genetic information into the host genome of dividing as well as non-dividing cells [16,17]. HIV-1 is the best studied lentivirus and most of the currently used lentiviral vectors (LVs) are based on its sequence [16,18–20]. Previous studies demonstrated that LVs allow for an efficient gene transfer in ES cells [21,22]. In addition, LVs have already been applied in first clinical gene therapy trials (e.g. [22–24]).

In the present study, we utilized LVs for the genetic modification of ES and iPS cells of mouse. To enable TK expression in undifferentiated pluripotent stem cells only, different promoters of pluripotency genes were used including Oct-3/4 [25,26], Nanog [11,27,28], EOS-C3 [29] or EOS-S4 [29]. Cells expressing TK are sensitive to GCV treatment. Using this approach, we successfully eliminated undifferentiated cells *in vitro*. However, to obtain a pure population of TK expressing ES cells, a hygromycin resistance cassette had to be incorporated in the LV. This led to elimination of undifferentiated cells after hygromycin pre-selection. Most importantly, *in vivo* transplantation of these LV transduced pre-selected ES cells led to loss of teratoma formation upon GCV administration to the mice.

## Materials and Methods

### Cell lines and cell culture

We used the murine ES cell line (α-PIG) carrying the puromycin resistance and eGFP cDNAs connected via an IRES (internal ribosomal entry site) element under control of the cardiac specific α-myosin heavy chain promoter. For undifferentiated conditions, ES cells were cultured on tissue plates or flasks coated with a layer of mitotically inactivated murine fibroblast cells (feeder cells) in DMEM supplemented with nonessential amino acids (0.1 mM), L-glutamine (2 mM), penicillin (100 units/ml), streptomycin (100 µg/ml), β-mercaptoethanol (0.1 mM), leukemia inhibitory factor (LIF) (ESGR) (500 units/ml), and fetal calf serum (FCS) (15% (v/v)).

For analysis of ES cell survival under undifferentiation conditions, cells were transduced with LVs (see below) and treated with or without GCV. Surviving undifferentiated cells were manually counted using three different fields of view that were counted twice.

For differentiation of ES cells into embryoid bodies (EBs) the mass culture protocol was used [30]. Briefly, lentiviral transduced or untransduced ES cells were split to single cells in differentiation medium and cell suspension was incubated at 37°C with ~80 rpm shaking for 14 days. The differentiation medium contained nonessential amino acids (0.1 mM), L-glutamine (2 mM), penicillin 100 (units/ml), streptomycin (100 µg/ml), β-mercaptoethanol (0.1 mM) and fetal calf serum (20% (v/v)) in IMDM (Gibco). GCV treatment was started after day 8. For analysis of ES cell survival under differentiation conditions, EBs were dissociated and stained as mentioned below (Immunocytochemistry). Oct3/4 positive cells were manually counted using one to three different fields of view that were counted five times, respectively.

For *in vivo* ES cell injection, undifferentiated ES cells were trypsinized and incubated on 10 cm tissue plates coated with 0.1% gelatin for 30 minutes. This enables a separation of feeder cells (reattachment on the plate) and ES cells (remaining in suspension). Afterwards, 1x10^6^ ES cells/ 100 µl suspension were prepared in DMEM without LIF and kept on ice before ES cell injection.

The murine iPS cell line used was provided by P. Sasse (University of Bonn, Germany) [31]. The murine iPS-Oct-GFP cell line was a gift from H. R. Schöler (Max Planck Institute for Molecular Biomedicine, Münster, Germany) and contains an eGFP expression cassette driven by the promoter of the pluripotency gene Oct3/4 leading to eGFP expression only in undifferentiated cells. iPS cells were cultured under undifferentiated conditions on tissue plates or flasks coated with a layer of feeder cells in DMEM supplemented with nonessential amino acids (0.1 mM), penicillin (100 units/ml), streptomycin (100 µg/ml), β-mercaptoethanol (0.1 mM), LIF (1.000 units/ml), fetal calf serum (FCS) (15% (v/v)), CHIR99021 (3µM; GSK-3 Inhibitor) and PD325901 (4µM; MEK1/2 Inhibitor).

### Cloning of LV constructs

The Nanog promoter including enhancer element was amplified from mouse genomic DNA (gDNA), isolated from mouse’s tail, using the following primers: 5’-cgtgatGTCGACAATTTCTTCTTCCATTGCTTAGACGG-3’ (forward) and 5’-tgcgccGGATCCAAGGGATTTCTGAAAAGGTTTTAGGC-3’ (reverse) [32] with *Sal*I and *BamH*I restriction sites (written in italic letters). Additional nucleotides (depicted in lowercase) were included to facilitate digestion of DNA by the restriction enzyme. The Oct-3/4 promoter including the proximal and distal enhancer was amplified from mouse gDNA (isolated from mouse’s tail) using the following primers: 5’-ttcattATCGATTCTAGGCACGCTTAGGGC-3’ (forward) and 5’-ttcattAGATCTCCGAGCCGGGGGCCTGGTGG-3’ (reverse) [32,33] with *Cla*I and *Bgl*II restriction sites (in italic letters) and additional nucleotides (again written in lowercase). EOS-C3 and EOS-S4 promoters were amplified from plasmids: PL-SIN-EOS-C(3+)-EiP and PL-SIN-EOS-S(4+)-EiP [29] using forward primer 5’-ggaaATCGATTTTATCCAGCCCTCACTCCT-3’ and reverse primer 5’-aattGGATCCTGGCTTTACCAACAGTACCG-3’ with *Cla*I and *BamH*I restriction sites (in italic letters) and additional nucleotides (in lowercase). The four promoters of pluripotency genes were cloned into the *Sal*I and *BamH*I or *Cla*I and *BamH*I site of the 3^rd^ generation LV plasmid pRRLSIN.cPPT.eGFP. WPRE [18,34]. Thereafter, the eGFP-cDNA was replaced by the TK cDNA of herpes simplex virus which was amplified from pGEM-TK containing TK cDNA originally obtained from Promega using primers 5’-gattGGATCCATGGCTTCGTACCCCTGCCA-3’ (forward) and 5’-aattCGCGAGTCAGTTAGCCTCCCCCA with *BamH*I and *Xho*I restriction sites (in italic letters) and additional nucleotides (in lowercase). For generation of double cassette LV construct STPH, the expression cassette for the hygromycin-resistance cDNA under control of the PGK promoter was cloned into the LV construct EOS-S4-TK (ST) between the TK cDNA and the WPRE element. The Hygromycin resistance cDNA was amplified from plasmid pPWL512 [35] containing hygromycin resistance gene using following primers: 5’-ttggGGATCCAGCCGCCACCATGAAAAAGC-3’ (forward) 5’-ggttCTCGAGATCGATCTATTCCTTTGCCCTCGGACGAGTGC-3’ (reverse) with *BamH*I and *Xho*I-*Cla*I restriction sites (in italic letters) and additional nucleotides (in lowercase).

### Production of LVs

The production of LVs was performed as previously described [36]. Briefly, LV plasmids as well as the structure and packaging plasmids pMDLg/pRRE [37], RSV-rev [37] and pMD2.G [18] were co-transfected into HEK 293T cells (ATCC) seeded on Poly-L-lysine coated 150-mm^2^ dishes. Cells were incubated at 37°C and 3% CO_2_ overnight. Medium was changed 12 hours later. 48 hours and 72 hours after transfection, the released viruses in the supernatant were collected and cell debris was removed using a SFCA bottle-top filter. Supernatant was centrifuged by an ultracentrifuge with SW32Ti rotor at 61,700g at 17°C for 2 hours. Subsequently, supernatant was discarded and virus pellet was resuspended in Hank’s Balanced Salt Solution (HBSS), respectively. Combined virus suspensions were concentrated by centrifugation over a 20% (w/v) sucrose cushion in a SW55Ti rotor at 53,500g at 17 °C for 2 hours. Finally, viruses were dissolved in HBSS and stored at -80 °C. The titers of the virus preparations were determined by measuring the amount of active viral reverse transcriptase (RT) using a colorimetric RT-ELISA (Roche) with a calibration curve.

### LV transduction of ES cells

1x 10^4^ ES cells were seeded in a 24-well plate with 1.2x10^5^ feeder cells/well (see also section cell lines and cell culture). After ES cells attached on the feeder cells, medium was removed and replaced with 300 µl of medium containing LVs overnight. Next day, medium was replaced by fresh medium and ES cells were further cultured for experiments. Before complete confluence, the ES cells were split according to experimental procedures. 

### Determination of provirus integration per genome using quantitative Realtime PCR (qPCR)

Genomic DNA (gDNA) of ES cells was isolated by classic phenol-chloroform extraction. After isolation, DNA was digested with *BamH*I and *EcoR*I to obtain smaller DNA fragments that are more suitable as template in qPCR. The qPCR analysis for quantification of provirus integration was performed using the IQ5 Real-time PCR System (Bio-Rad) and the TaqMan method (iQ Multiplex Powermix, Bio-Rad) in accordance to the manufacturer’s instruction. To detect the amount of integrated provirus DNA, Late-RT primers 5’- TGTGTGCCCGTCTGTTGTGT -3’ (forward), 5’- GAGTCCTGCGTCGAGAGAGC -3’(reverse) and the 5’-FAM-labeled probe 5’-FAM-CAGTGGCGCCCGAACAGGGA-BHQ1-3’ were used [38]. Primers and the Texas Red labeled probe of the house keeping gene Burkitt lymphoma receptor 1 (BLR1) were used to detect total gDNA as described elsewhere [39]. Since BLR1 is a single-copy gene, it can be used to determine absolute copy number per genome by comparison of the cycle threshold (Ct) values from virus specific probe versus the BLR1 probe. Copy numbers were evaluated by following formulas: Copy number/genome = 2^ΔCt^ with ΔCt = Ct (BLR1) -Ct (LateRT).

### Generation and analysis of ES cell clones

1x10^4^ ES cells were seeded on feeder cells in a 24 well plate and transduced with lentiviral vectors (NT or OT). 72h after transduction ES cells were trypsinized and 500 ES cells were seeded on 100-mm tissue dishes coated with feeder cells (4x10^6^ feeder cells per dish). 7 days later, ES cell colonies were scraped carefully with a pipette tip to dislodge the colonies from the feeder cell layer. Subsequently, ES cell colonies were transferred into a well of a 24-well plate containing 120µl 0.05% trypsin/EDTA. After incubation at 37°C for 5 minutes, the cells were resuspended gently to dispense to single cells and split on three new 24 wells and further cultivated until they reached confluency. When reaching confluency, one well was used for freezing, one well for further cultivation and subsequent experiments and one well for gDNA isolation by classic phenol-chloroform extraction. After isolation, gDNA was digested with *Bam*HI. Digested gDNAs of different clones were loaded on an agarose gel and further tested by using Southern Blot procedure with lentiviral integration specific probe (WPRE probe, amplified from a lentiviral construct using the following primers: 5’-GGACGAACATGGCGTGAAGC-3’ (forward) and 5’-GGACGTCCCGCGCAGAAT-3’ (reverse)). Experiments presented in the manuscript were performed with four clones (NT #8, NT #11, OT #4, OT #11) with only one integrant (one band on southern blot).

### Quantification of mRNA level using qPCR

Total RNA of ES cells was extracted by Guanidine-isothiocyanate/phenol method using peqGOLD Tris-FAST (peqLAB). RNA was transcribed into cDNA using transcriptor first-strand cDNA synthesis kit (Roche). mRNA level was detected by application of the iQ SYBR Green Supermix (Bio-Rad) with specific primers for TK (5’-GATGACTTACTGGGCAGGTG-3’ (forward) and 5’-GATGGCGGTCGAAGATGAG (reverse). mRNA level was normalized by applying murine GAPDH primers 5’-CCACTCACGGCAAATTCAAC-3’ (forward) and 5-‘GTTCACACCCATCACAAACATG-3’ (reverse)

### LDH assay

The effect of GCV treatment of TK transduced cells on cytotoxicity was analyzed using the LDH assay (Roche). The LDH in cell culture supernatants released from dead cells was either directly analyzed using LDH assay or LDH was measured after complete lysis of cells of each set of samples: 3x10^3^ ES cells transduced with NT, OT, CT and ST or untransduced cells were seeded on a 24-well plate coated with a layer of feeder cells. One day after ES cell seeding, the medium was replaced by 500 µl fresh medium with 20 µM GCV. 36 hours later, further 500 µl fresh medium with 20 µM GCV was added on the cells. Further 36 hours later (=72 hours with GCV treatment), 50 µl of the supernatants from ES cells with GCV treatment and ES cells with GCV treatment after adding of lysis buffer for 15 minutes were each analyzed using the LDH assay in accordance to the manufacturer’s instructions. To obtain “background” signals feeder cells without ES cells were seeded as well and treated with GCV. 50 µl of the supernatants from feeder cells alone and feeder cells alone after adding of lysis buffer for 15 minutes were analyzed. These values with or without lysis were each subtracted from the extinction values obtained from ES cells. The differences between values with or without lysis represent the number of ES cells that survived GCV treatment. Extinction was each measured using ELISA reader (TECAN).

The assay was applied for the quantification of ES cell survival under non-differentiation conditions.

### Injection of ES cells in murine hind limbs

100µl cell suspension with 1x10^6^ ES cells (see also section cell lines and cell culture) were injected s.c. into the hind limbs of 4 to 8 weeks old Fox, Chase SCID® Beige mice (Charles River). Three hours or seven days after ES cell injection, mice were administered i.p. with either saline solution (0.9% (w/v) NaCl) or GCV (20 mg/kg/day) for 12 days. After treatment, mice were sacrificed for the analysis of potential teratoma formation. The teratomas were analyzed by measuring their weight and size. For further analysis, teratomas were fixed in 4% (w/v) paraformaldehyde (PFA) in phosphate-buffered saline (PBS), embedded in O.C.T. (Tissue-Tec) and stored at -80 °C. Sections were prepared in 10 µm slices for H&E staining.

All experiments involving animals were done in accordance with the German legislation on protection of animals and the NIH Guidelines for the Care and Use of Laboratory Animals. The study was approved by the Landesamt für Natur, Umwelt und Verbraucherschutz Nordrhein-Westfalen, Germany. Cell injection was performed under isofluran inhalation anaesthesia, and all efforts were made to minimize suffering.

### Hematoxylin and eosin stain

Teratoma sections were incubated in PBS for 5 minutes two times. Afterwards, slides were treated with hematoxylin (Mayers hemalaun, Merck) for 1 minute and then washed in fluent water for 5 minutes. After that, slides were stained with eosin (Eosin G, Merck) for 30 seconds and washed in fluent water for 5 minutes. Finally, slides were mounted with Roti®-Histokitt (Carl Roth). Images were taken using an AxioStart (Carl Zeiss) and AxioCam (Carl Zeiss).

### Immunocytochemistry

EBs (see also section cell lines and cell culture) were collected and washed twice with PBS. After that, EBs were incubated in Collagenase B (1 mg/ml) (Roche) for 20 minutes in a shaker with 600 rpm at 37°C. 3x10^5^ single cells from dissociated EBs were seeded in a 24-well plate with cover slips which were coated with 0.1% (w/v) gelatin. After reattaching of the cells on the cover slips (24 hours later), cells were fixed with 4% (w/v) PFA. Cells fixed on the cover slips were incubated with primary antibodies in solution containing 0.05% (v/v) donkey serum (Jackson ImmunoResearch), 0.1% (v/v) triton, anti Oct-3/4 (1:100, Santa Cruz Biotechnology) and anti α-actinin (1:400, Sigma-Aldrich) in PBS for 2 hours. After removing the primary antibodies and washing three times with PBS, cells were further incubated with secondary antibodies containing anti Dylight 549 and anti Dylight 649 (both 1:400, Jackson ImmunoResearch) in Hoechst stain solution (Sigma-Aldrich) for 1 hour. Images were taken using Axio Observer. Z1 with ApoTome system (Carl Zeiss) and AxioCam MRC5 (Carl Zeiss).

### Statistical analysis

All values mentioned in the text are given as mean±SEM. Statistical significance was determined by one-way analysis of variance (ANOVA) or by using Student’s *t*-test. We considered p<0.05 as statistically significant.

## Results

### I: LV-mediated TK expression in pluripotent stem cells and GCV treatment

In the present study, we used a murine ES cell line that carries the cDNAs for puromycin resistance and green fluorescent protein (eGFP) connected via the IRES (internal ribosomal entry site) element. Transgene expression is driven by the cardiac specific α-myosin heavy chain (α-MHC) promoter. Thereby, successful differentiation of ES cells can be assessed by fluorescence microscopy after applying culture conditions that favor cardiac differentiation [40].

**Figure 1:**
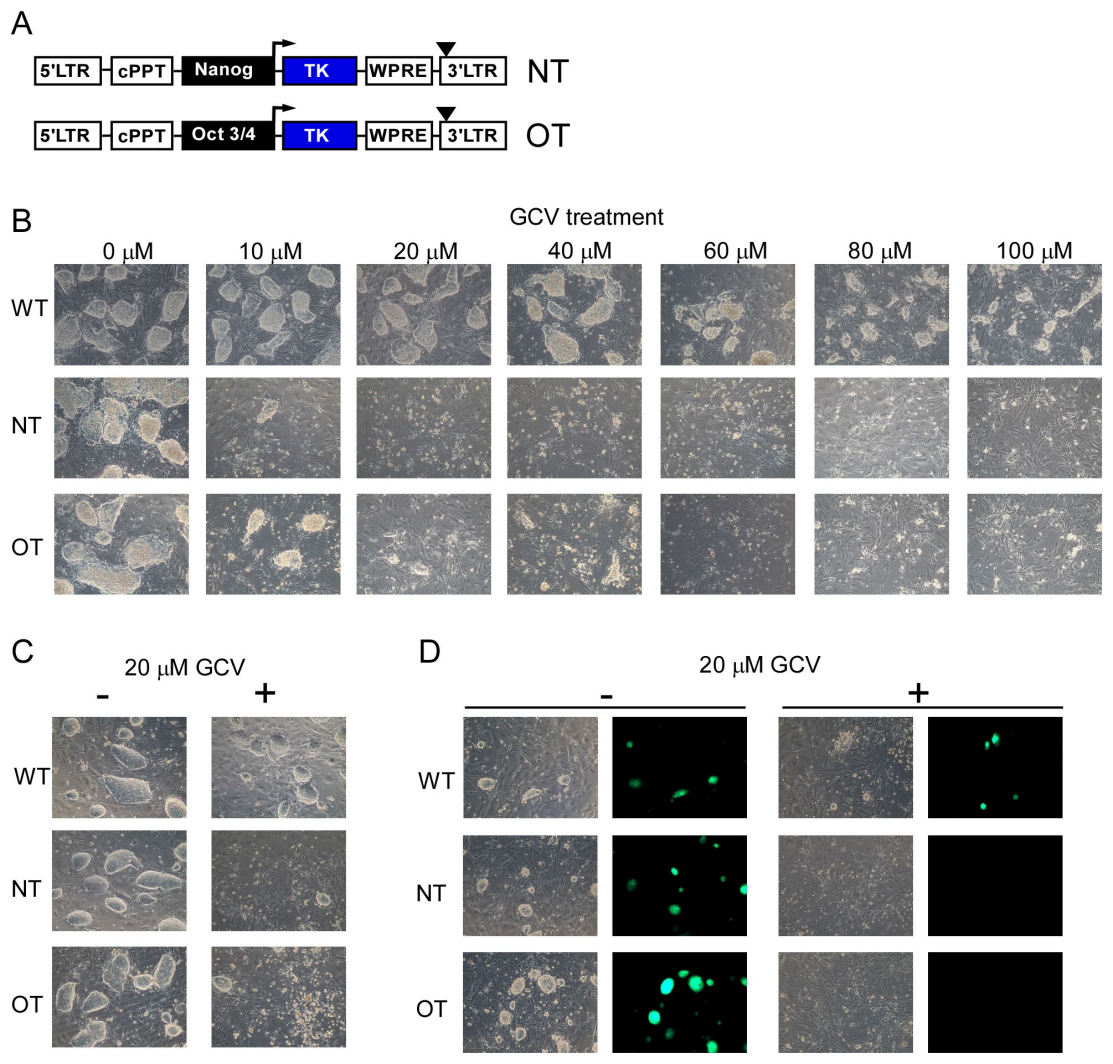
Constructs of LVs carrying TK expression cassette and analysis of NT- or OT-transduced ES and iPS cells *in vitro*. (**A**) Constructs of LVs carrying thymidine kinase (TK) cDNA from herpes simplex virus driven by promoters of pluripotency genes Nanog (NT) or Oct-3/4 (OT). (**B**) ES cells were transduced with LVs (NT or OT, 300 ng of reverse transcriptase) or not transduced (WT) and further treated with 0, 10, 20, 40, 60, 80 or 100 µM GCV for 72 hours. Representative brightfield images are shown (n=3). (**C**) and (**D**) iPS cells (C) or iPS-Oct-GFP cells (D) were transduced with LVs (NT or OT, 300 ng of reverse transcriptase) or not transduced (WT) and further treated with (+) or without (-) 20 µM GCV for 72 hours. Representative brightfield images (C, D) and fluorescence images (D) are shown (n=3).

In order to generate ES cells stably expressing TK only in undifferentiated cells [25–28], we first applied LVs carrying HSV-TK cDNA under control of promoters of the pluripotency genes Nanog or Oct-3/4 (NT and OT) (Figure 1A). Indeed, treatment of the transduced cells with GCV for 72 hours had a toxic effect on undifferentiated ES cells (Figure 1B). Using different concentrations of GCV, we found that treatment with 20 µM GCV was sufficient to eliminate undifferentiated cells but had no adverse effect on wildtype ES cells (Figure 1B). Therefore, 20 µM GCV was used in all further *in vitro* experiments.

As iPS cells also carry the potential risk of tumor formation after transplantation in *in vivo* applications, removal of iPS cells that did not undergo differentiation is an important task. Thus, we tested the NT- and OT-LVs in two different mouse iPS cell lines: iPS and iPS-Oct-GFP, the latter containing an eGFP expression cassette under control of promoter of the pluripotency gene Oct-3/4. Upon GCV administration, death of NT- and OT-transduced iPS-cells could be observed (Figure 1C). Analysis of Oct-3/4 promoter driven eGFP expression in iPS-Oct-GFP cells also revealed efficient killing in the NT- or OT-transduced cells after GCV treatment (Figure 1D). Taken together, besides murine ES cells, the TK/GCV system also led to an efficient elimination of undifferentiated iPS cells *in vitro*.

### II: LV mediated TK expression in mixed ES cell populations

**Figure 2:**
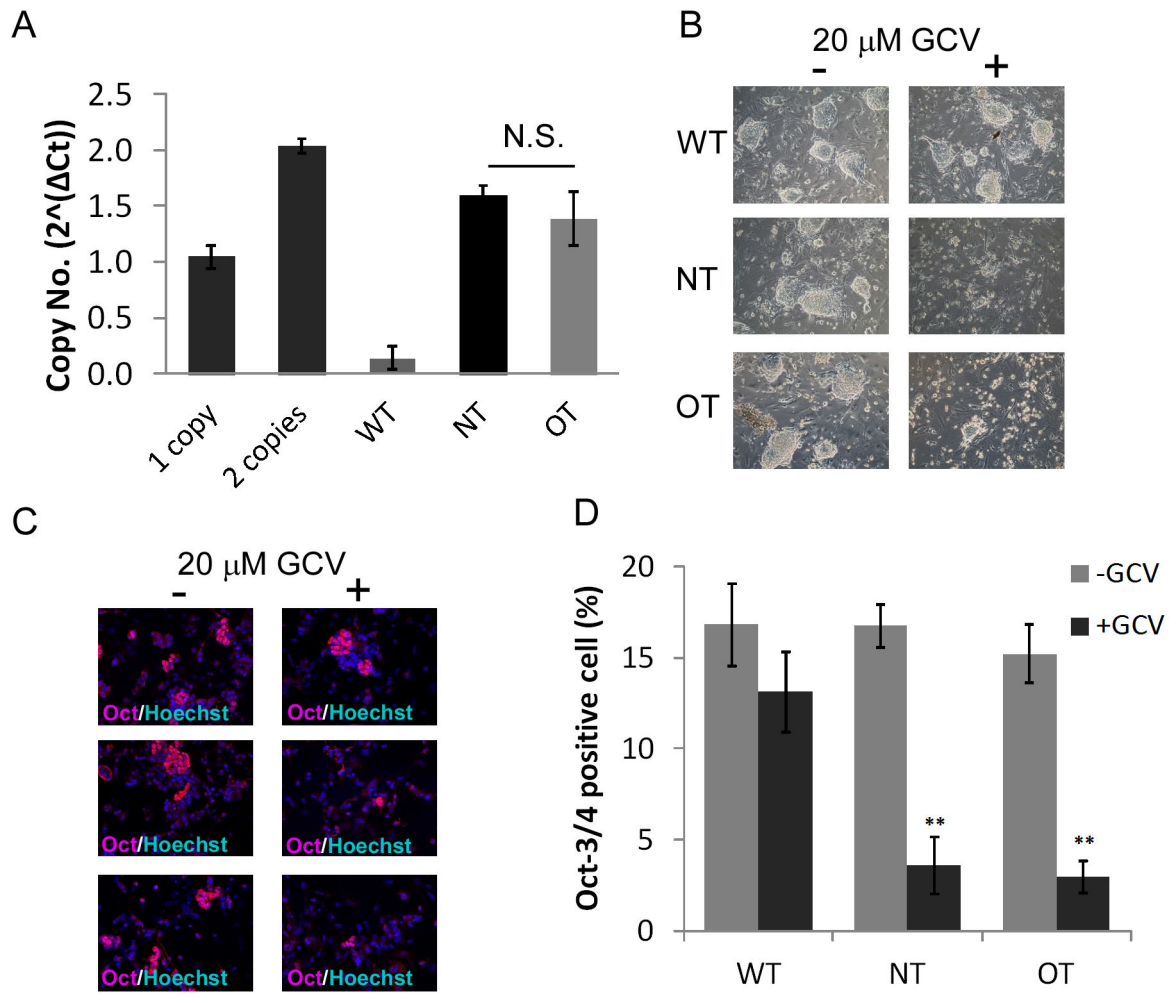
Analysis of mixed ES cell populations transduced with lentiviral NT or OT with low copy number. (**A**) ES cells were transduced with different amounts of lentiviral NT or OT or were not transduced (WT). The copy number of LVs in mixed ES cell populations was analyzed by qPCR. Shown are the results of mixed ES cell populations carrying ^≈^1.5 copy numbers per genome in average (n=3, Mean±SEM; N.S., not significant, ANOVA). As control, two transgenic mice previously analyzed by Southern Blot to have one or two integrants (data not shown), were also analyzed by qPCR. (**B**) ES cells were transduced with NT or OT (1.5 copy numbers per genome in average) or not transduced (WT) and treated with (+) or without (-) 20 µM GCV. Representative brightfield images are shown (n=3). (**C**) The untreated ES cell populations shown in (B) were differentiated as EBs with (+) or without (-) 20 µM GCV treatment. After 14 days of differentiation dissociated EBs were immunostained with Oct-3/4 (red) and Hoechst (blue) indicating Oct-3/4-positive cells (ES cells) and nucleus, respectively. Representative images are shown (n=3). (**D**) Percentage of Oct-3/4-positive cells on day 14 of differentiation with and without GCV treatment analyzed by manual counting of Oct-3/4-positive cells on images representatively shown in (C) using three different fields of view that were counted five times (Mean±SEM, **P<0.01, ANOVA).

For clinical applications, it is desired to use a low copy number of LVs to minimize the potential risk of insertional mutagenesis by vector integration [41]. Therefore, we investigated ES cell populations with a low average copy number (e.g. one to two provirus integrations) per genome. A recent report showed that quantitative real time PCR (qPCR) can be used as a reliable method to determine the copy number of LVs [39]. Hence, we transduced ES cells with different LV concentrations and analyzed their average integration number using qPCR. These LV transduced ES cell populations without additional selection are further referred to as mixed ES cell populations. Mixed ES cell populations with an average copy number of 1.5 provirus integrations per genome (1.60±0.09 (NT) and 1.38±0.24 (OT)) were chosen for further analysis (Figure 2A). GCV treatment of these undifferentiated populations inhibited cell survival as shown by bright field images (Figure 2B). Next, we investigated the effect of GCV in mixed ES cell populations under differentiation conditions. We differentiated untransduced as well as NT- or OT-transduced mixed ES cell populations using the mass culture protocol [30] for 14 days. The resulting embryoid bodies (EBs) were dissociated and characterized by immunostaining with antibodies against Oct-3/4 (pluripotency marker) and Hoechst (nuclear stain) (Figure 2C). Without GCV treatment similar percentages of Oct-3/4-positive cells were observed in untransduced (16.8%±2.3) as well as NT- (16.8%±1.2) or OT-transduced (15.2%±1.6) mixed ES cell populations indicating that cells remained undifferentiated and maintained their pluripotent status (Figure 2D). In the untransduced ES cells, we observed 13.1%±2.2 Oct-3/4 positive cells after GCV treatment indicating that GCV alone had no significant effect on cellular vitality (Figure 2D). In contrast, only 3.6%±1.6 and 3.0%±0.9 of the NT- or OT-transduced mixed ES cell populations were Oct-3/4-positive when treated with GCV (Figure 2D). Thus, most of the undifferentiated ES cells are eliminated during differentiation in the presence of NT- or OT-driven TK expression.

Differentiation of ES cell-derived cardiomyocytes was not influenced neither by the LV transduction nor the GCV treatment alone as shown by cardiac-specific α-MHC promoter driven eGFP expression (Supplementary Figure S1).

Taken together, ES cells transduced with NT- and OT-LVs at low copy numbers exhibited high killing efficiency upon GCV treatment after differentiation. Nevertheless, the mixed populations still contained around 3 to 4% Oct-3/4 positive cells on day 14 post differentiation (Figure 2D) suggesting a potential risk of teratoma formation *in vivo*. To address whether higher levels of TK expression lead to more efficient elimination, we tried to increase TK abundance by using other promoters.

### III: LV mediated TK expression in mixed ES cell populations using EOS promoters

**Figure 3:**
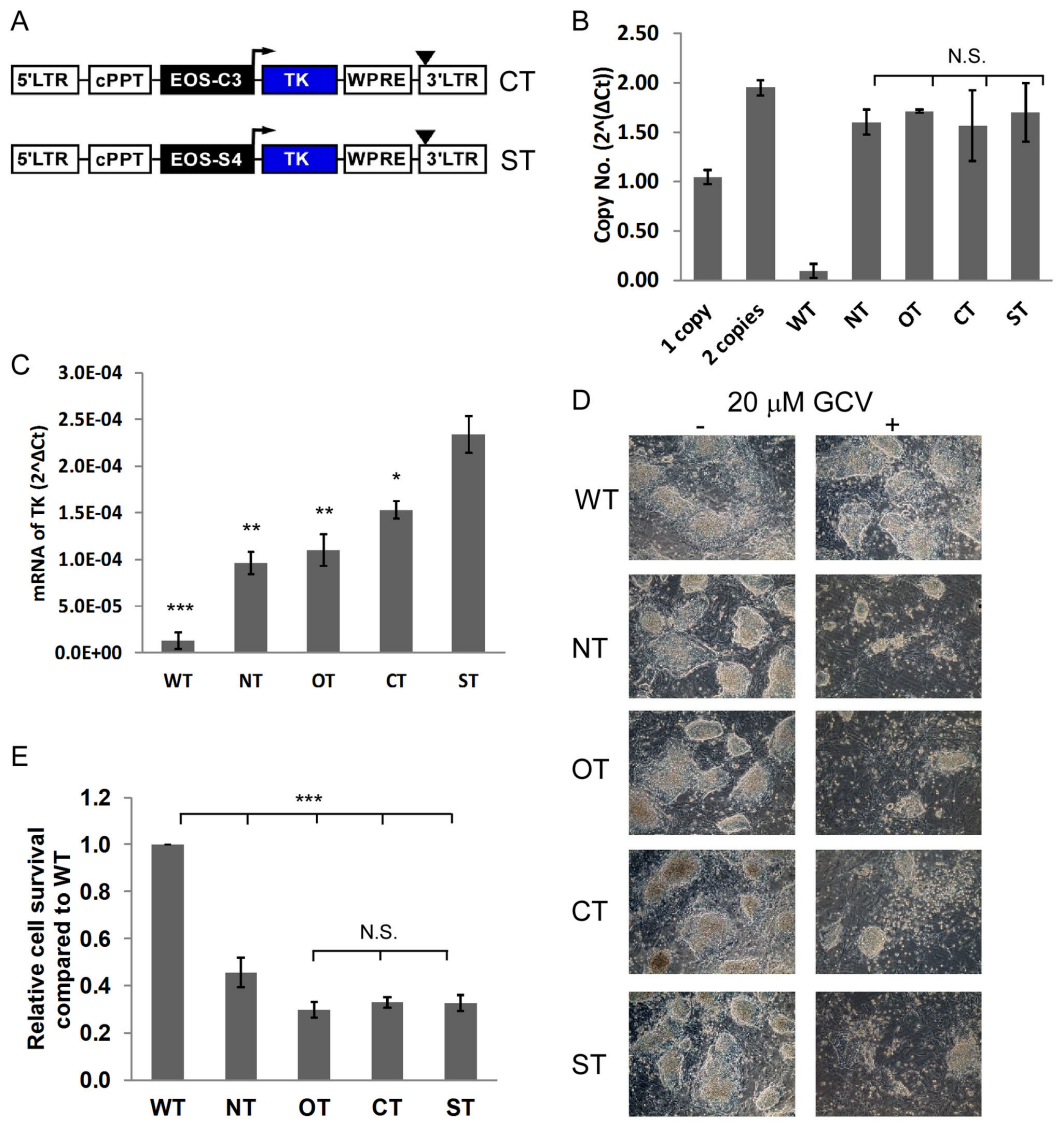
Analysis of ES cells transduced with LVs using different promoters of pluripotency genes. (**A**) In addition to NT and OT (Figure 1A) LVs carrying TK cDNA under the control of the EOS-C3 (CT) and EOS-S4 (ST) promoters were constructed. (**B**) The copy number of LVs in mixed ES cell populations transduced with NT, OT, CT and ST or not transduced (WT) was analyzed by qPCR (n=3, Mean±SEM, N.S., not significant, ANOVA). As control, DNAs of two transgenic mice were used, that were previously analyzed by Southern Blot to have one or two integrants. (**C**) TK expression on mRNA level of NT, OT, CT and ST transduced ES cells (1.5 copy numbers per genome in average) or not transduced (WT) was analyzed by qPCR and normalized to GAPDH (n=3, Mean±SEM, *P<0.05, **P<0.01, ***P<0.001 compared to ST, ANOVA). (**D**) Brightfield images of NT, OT, CT, ST (1.5 copy numbers per genome in average) or not transduced (WT) ES cells after treatment with (+) or without (-) 20 µM GCV for 72 hours. Representative images are shown (n=4). (**E**) ES cells from (D) with GCV treatment were analyzed with LDH assay as described in experimental methods section. Shown are the relative numbers of NT, OT, CT or ST transduced ES cells that survived GCV treatment as compared to untransduced (WT) ES cells (n=3, Mean±SEM; ***P<0.001, compared to WT; N.S., not significant, ANOVA).

In order to achieve higher levels of TK expression, we applied the promoters of the pluripotency genes EOS-C3 and EOS-S4, shown to drive stronger expression than Nanog or Oct-3/4 promoters in human or murine ES and iPS cells [29]. The promoters were cloned into the LV backbone leading to LV-constructs EOS-C3-TK (CT) and EOS-S4-TK (ST) (Figure 3A). The four LVs (NT, OT, CT, ST) were used in parallel for transduction of murine ES cells and LV integration was determined by qPCR, respectively. Mixed ES cell populations with an average copy number of approximately 1.5 per genome were further investigated (Figure 3B). TK expression on mRNA level was analyzed using qPCR (Figure 3C). These experiments revealed that both, EOS-C3 and EOS-S4, promoters gave rise to higher TK expression levels compared to Nanog and Oct-3/4 in murine ES cells (Figure 3C). However, the highest TK abundance was detected using EOS-S4 promoter (Figure 3C). We observed a clear reduction of surviving ES cells in all four cell populations after GCV treatment (Figure 3D). Next, we analyzed the TK/GCV induced killing efficiency by detecting Lactate Dehydrogenase (LDH) that is released by dead cells into the cell culture supernatant. Interestingly, a higher TK expression level did only slightly increase the effect of GCV on ES cells (Figure 3E) demonstrating that the portion of surviving cells seems to be the consequence of incomplete transduction of the ES cell population.

### IV: LV mediated TK expression in single integrant ES cell clones and GCV treatment

**Figure 4:**
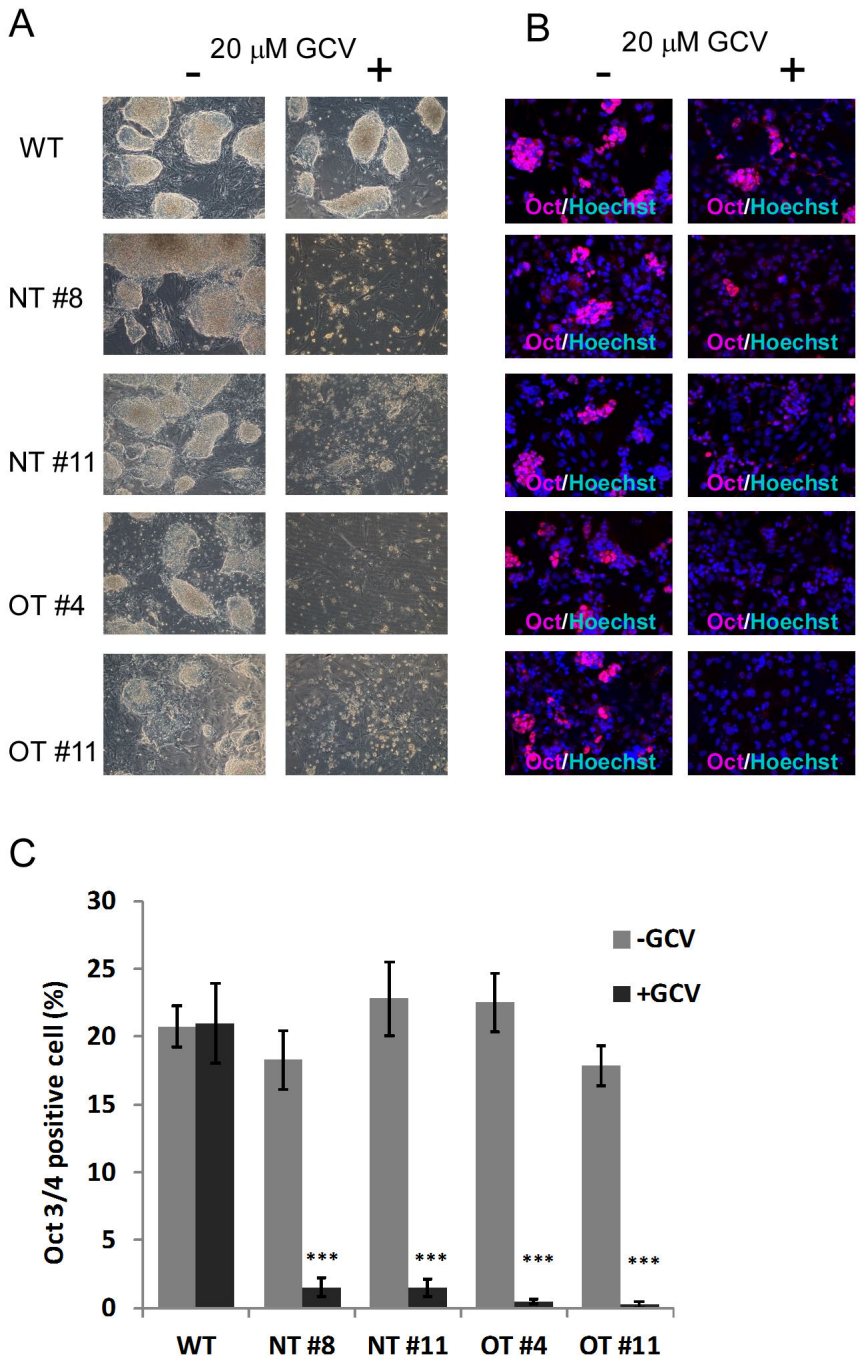
Analysis of single LV-integrant NT- or OT-transduced ES cell clones *in vitro*. (**A**) ES cells were transduced with NT or OT and picked ES cell clones carrying one integrant (NT #8, NT #11, OT #4, OT#11) or not transduced ES cells (WT) were treated with (+) or without (-) 20 µM GCV for 72 hours. Representative brightfield images are shown (n=3). (**B**) The untreated ES cell clones shown in (A) were differentiated as EBs with (+) or without (-) 20µM GCV. After 14 days of differentiation dissociated EBs were immunostained with Oct-3/4 (red) and Hoechst (blue) indicating Oct-3/4-positive cells (ES cells) and nuclei, respectively. Representative images are shown (n=3). (**C**) Percentage of Oct-3/4-positive cells of dissociated EBs on day 14 after differentiation with and without GCV treatment analyzed by manual cell counting of images shown in (B) by using one field of view that was counted five times (Mean±SEM; ***P<0.001 compared to without GCV, respectively, ANOVA)..

The mixed ES cell populations analyzed so far still resulted in incomplete elimination of undifferentiated cells after GCV treatment. However, a few pluripotent cells could lead to formation of teratomas *in vivo* [42]. To study whether a complete abolishment of undifferentiated cells could be reached in cells carrying one integrant, we next analyzed clones derived from individual cells from single integrant NT or OT expressing ES cell lines. Single ES cell clones that carry only one integrant per genome were identified by Southern blot analysis (Figure S2A, B). Two different single-integrant clones of each NT- and OT-transduced ES cells were used for further investigations (NT #8, NT #11, OT #4, OT #11). Under undifferentiation conditions, cells of all four clones were eliminated upon GCV treatment (Figure 4A). The differentiation of the ES cells of all four clones into ES cell-derived cardiomyocytes was not influenced by the LV transduction or the GCV treatment (Figure S2C). Importantly, under differentiation conditions, only 1.5%±0.7 (NT #8), 1.5%±0.6 (NT #11), 0.5%±0.2 (OT #4) and 0.3%±0.3 (OT #11) of surviving Oct-3/4 positive cells were observed on day 14 post differentiation after GCV treatment for 6 days (Figure 4B,C). These data demonstrated a much higher efficiency of the TK/GCV system in single integrant ES cell clones compared to the mixed cell populations where 3 to 4% of Oct-3/4 positive cells were observed under differentiation conditions (see also Figure 2D). 

### V: LV mediated TK expression and GCV treatment in mixed ES cell populations using Hygromycin pre-selection

**Figure 5:**
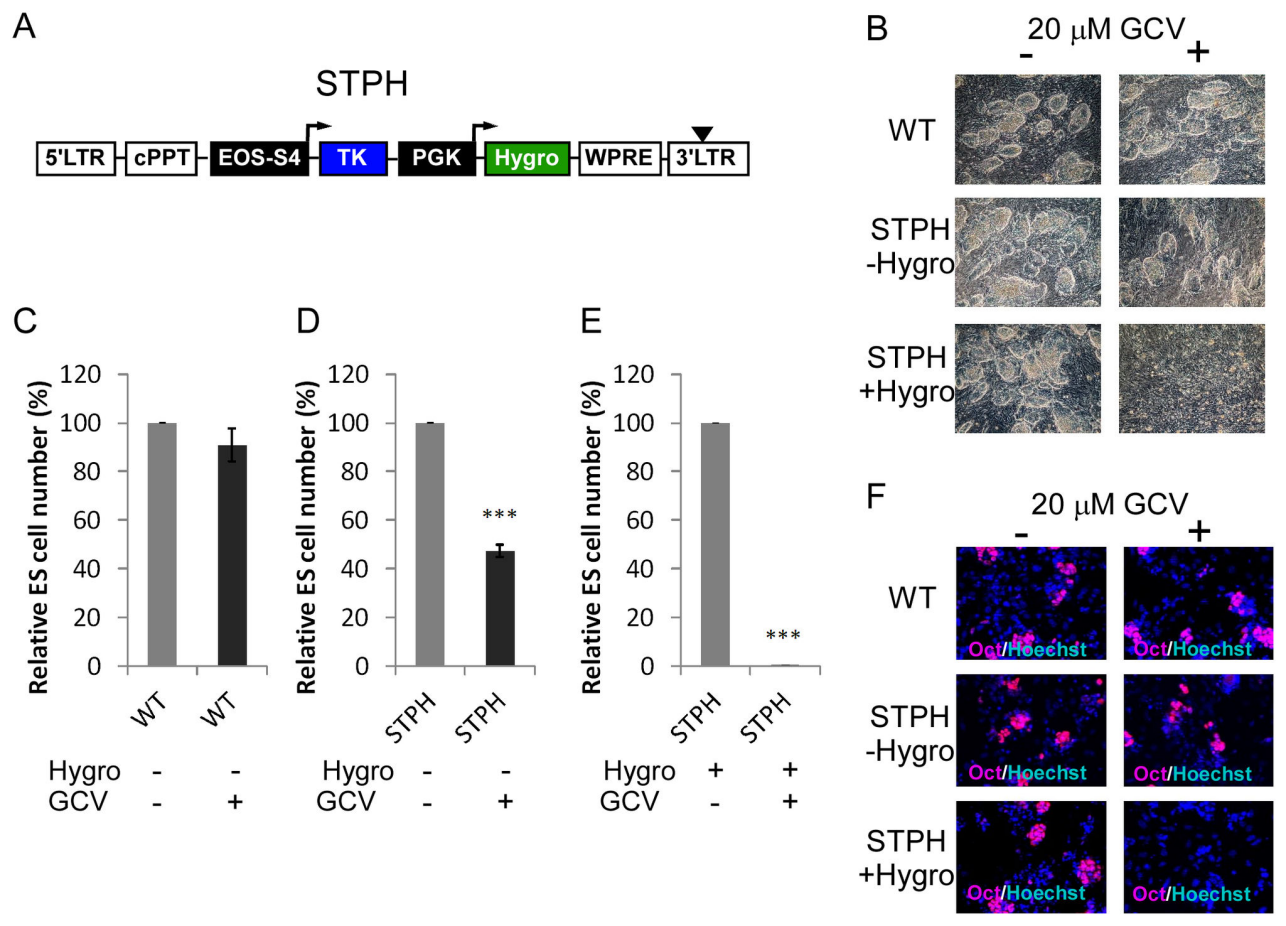
Analysis of ES cells transduced with LVs carrying a hygromycin resistance gene. (**A**) Construct of LV carrying additional hygromycin resistance gene driven by PGK promoter and cDNA of TK under control of EOS-S4-promoter (STPH). (**B**) ES cells were transduced with STPH (1.5 copy numbers per genome in average) or not transduced (WT) and treated with (+) or without (-) 20 µM GCV for 72 hours after pre-selection with (+Hygro) or without hygromycin (-Hygro). Representative brightfield images are shown (n=3). (**C**) (**D**) and (**E**) Relative cell survival of untransduced ES cells (WT) (C), STPH-transduced ES cells (1.5 copy numbers per genome in average) without pre-selection (D) and STPH-transduced ES cells (1.5 copy numbers per genome in average) with pre-selection (E) with (+) or without (-) 20 µM GCV treatment (n=3, Mean±SEM; ***P<0.001 compared to without GCV treatment, respectively, Student’s *t*-test). Data is based on images representatively shown in (B); undifferentiated cells were manually counted using three different fields of view that were counted twice. (**F**) The untreated ES cell populations shown in (B) were differentiated as EBs with (+) or without (-) 20 µM GCV. After 14 days of differentiation dissociated EBs were immunostained with Oct-3/4 (red) and Hoechst (blue) indicating Oct-3/4-positive cells (ES cells) and nuclei, respectively. Representative images are shown (n=3).

The use of single integrant NT- and OT-transduced ES cell clones resulted in a more efficient elimination of undifferentiated cells after GCV treatment (see also Figure 4C) as compared to mixed ES cell populations (see also Figure 2D). Nevertheless, as a complete abolishment of undifferentiated cells is required, these approaches are not efficient enough. For excluding the untransduced ES cells from mixed populations an antibiotic pre-selection scheme was chosen: We incorporated a hygromycin resistance gene expression cassette in the ST construct leading to construct EOS-S4-TK-PGK-Hygromycin (STPH) (Figure 5A). The hygromycin resistance gene was under the control of the ubiquitous PGK promoter enabling hygromycin selection of successfully transduced ES cells of each state.

The STPH vector was then used for ES cell transduction and mixed ES cell populations with an average copy number of 1.5 per genome were treated with GCV after hygromycin pre-selection for 9 days. The GCV treatment of the unselected, undifferentiated STPH-transduced ES cells resulted in a significant reduction of ES cells (47.3%±0.4 ES cell survival) whereas untransduced cells showed no difference with or without GCV as shown by brightfield images (Figure 5B) and by cell counting (Figure 5C, D). Importantly, with hygromycin pre-selection, only 0.4%±0.3 undifferentiated STPH-transduced ES cells were observed in the presence of GCV indicating pre-selection is required for almost complete elimination of ES cells (Figure 5B, E).

Furthermore, we tested if a shorter time span of hygromycin pre-selection is applicable and whether continuous hygromycin treatment is required. In addition to hygromycin selection for 9 days (Figure S3B see also Figure 5E), we tested pre-selection for 6 days followed by 3 days without selection and subsequent GCV treatment (Figure S3C) as well as 9 days pre-selection followed by 3 days without hygromycin before administration of GCV (Figure S3D).

Importantly, treatment for 9 days with hygromycin followed directly by GCV administration resulted in an identical ES cell survival as the hygromycin treatment for 9 days followed by 3 day incubation without selection and subsequent GCV administration (0.15%±0.08 vs. 0.15%±0.15, respectively). The results indicate that after hygromycin selection for 9 days hygromycin can be withdrawn before GCV selection. On the other hand, if the duration of hygromycin pre-selection is reduced to 6 days and cells are subsequently cultivated for 3 days without antibiotics, 3.66% ±0.63 of ES cells survive after GCV application. Consequently, shorter pre-selection seems not to be sufficient to ensure survival of only transduced cells. Taken together, after 9 days of pre-selection a subsequent removal of selection pressure does not alter the efficacy of GCV-mediated elimination of undifferentiated cells.

Alternatively, we speculated that an increase of lentiviral copy number might result in a similar efficiency: We transduced ES cells with higher LV concentrations of STPH leading to an average copy number of 3.8. As high copy numbers increase the risk of insertional mutagenesis we did not further raise the average copy number. GCV treatment of these high copy ES cells without pre-selection revealed strong elimination of undifferentiated cells (35.4%±2.0 ES cell survival) as shown by brightfield images (Figure S4A) and cell counting (Figure S4B, C). These data demonstrate that increasing the copy number significantly increases the elimination efficiency of TK/GCV system, but complete killing of undifferentiated cells was not achieved.

Importantly, when differentiating STPH-transduced ES cells with low copy number, that were pre-selected with Hygromycin for 9 days, we observed no Oct-3/4 positive cells after GCV treatment (Figure 5F).

In conclusion, STPH-transduced ES cells of low copy number with hygromycin pre-selection for 9 days resulted in successful elimination of ES cells upon GCV treatment. Although, increasing the lentiviral copy number showed lower cell survival of undifferentiated ES cells without pre-selection the complete elimination of ES cells was not achieved and thus, pre-selection seems to be necessary.

### VI: *In vivo* application of TK expressing ES cells

**Figure 6:**
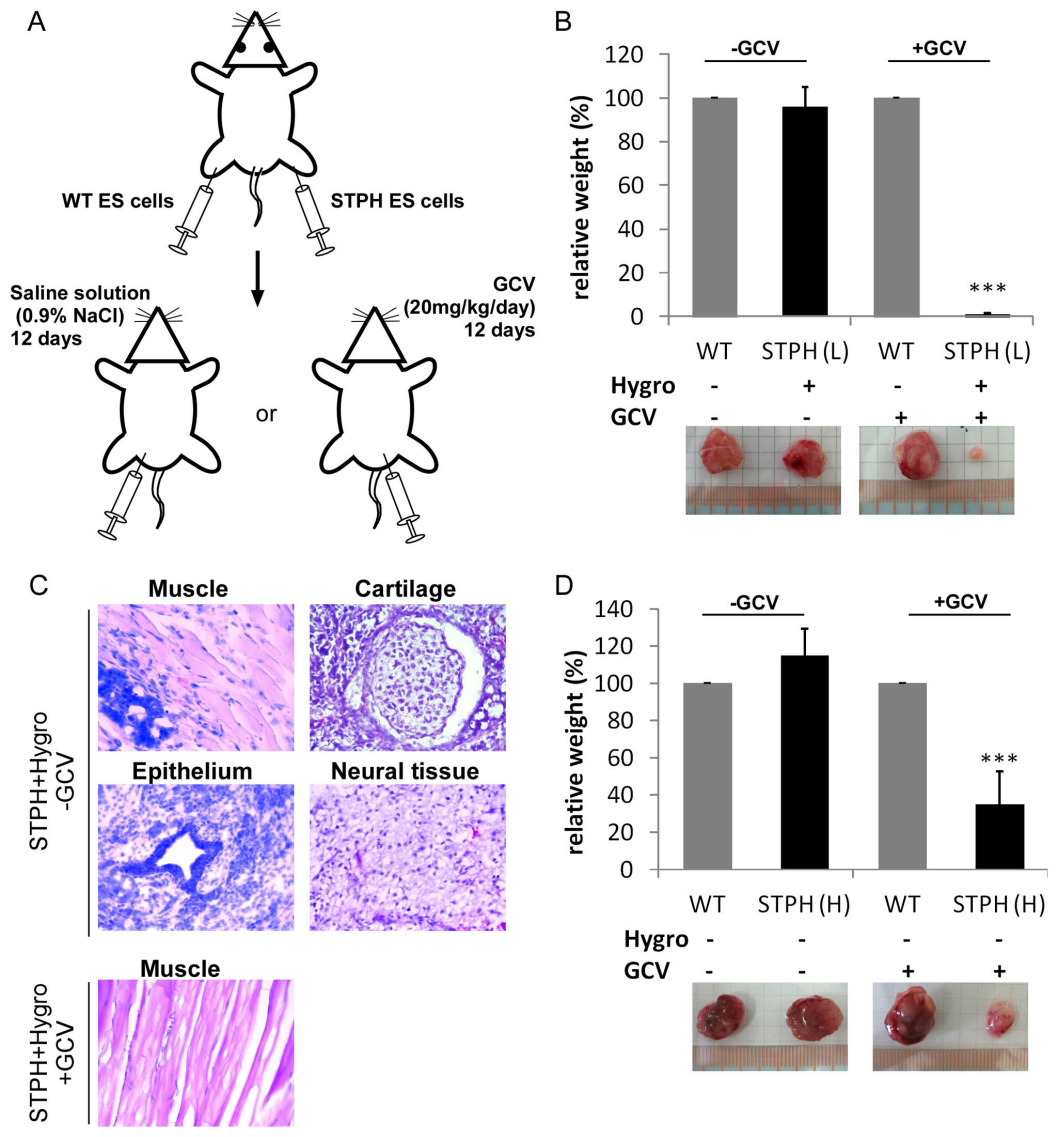
*In vivo* application of STPH-transduced ES cells. (**A**) Schematic illustration of *in vivo* procedure: 1 x 10^6^ ES cells (STPH-transduced or not transduced (WT)) were injected s.c. into hind limbs of SCID/beige mice. Saline solution (0.9% (w/v) NaCl) or GCV (20 mg/kg/day) were administrated i.p. for 12 days. (**B**) Relative weight of teratoma from *in vivo* application of hygromycin pre-selected STPH-transduced ES cells with low copy number (L (1.5 copy numbers per genome in average)) or untransduced ES cells (WT) with (+) or without (-) GCV treatment. Note, that only one out of six mice showed formation of tissue when applying STPH pre-selected cells and GCV administration (n≥4, Mean±SEM; ***P<0.001 compared to WT +GCV, ANOVA). (**C**) H&E stained sections of explanted teratoma emerging from injection of pre-selected (+Hygro) STPH-transduced ES cells (1.5 copy numbers per genome in average) without GCV (-GCV, upper) or with GCV treatment (+GCV, lower), demonstrating muscle, cartilage, glandular epithelium and neural tissue as indicated. Note, that only muscle tissue was detected in the single mouse that showed tissue development when applying STPH-transduced pre-selected cells and GCV treatment. (**D**) Relative weight of teratoma from *in vivo* application of STPH-transduced ES cells with high number (H (3.8 copy numbers per genome in average)) or untransduced ES cells (WT) without hygromycin pre-selection and with (+) or without (-) GCV treatment (n=5, Mean±SEM; ***P<0.001, compared to WT +GCV, ANOVA).

In the next step, we applied the LV mediated TK/GCV system tested so far *in vitro* in an *in vivo* mouse teratoma model to investigate tumor formation due to undifferentiated cells [42]. Therefore, we injected 1x10^6^ untransduced or STPH-transduced undifferentiated ES cells after hygromycin pre-selection s.c. into the left and right hind limbs of 8-week old SCID® mice, respectively (Figure 6A). Three hours after ES cell injection, we administered mice i.p. with either saline solution (0.9% (w/v) NaCl) or with 20 mg/kg GCV per day for 12 days. Teratoma will only form of undifferentiated cells that survived the selection.

Three weeks after ES cell injection mice were sacrificed for analysis of teratoma formation: No significant difference in the weight of teratoma was detected in mice treated with saline solution after injection of untransduced or STPH-transduced ES cells after pre-selection (Figure 6B). In addition, the average size of teratomas (maximum dimension) was more than 1 cm, respectively (Table 1). Sections of the teratomas exhibited tissues from all three germ layers as representatively shown for STPH-transduced and pre-selected ES cells without GCV treatment (Figure 6C upper). The same results for the teratoma formation were obtained after injection of untransduced cells and treatment with GCV (Figure 6B and Table 1) indicating no influence of GCV or hygromycin alone. Most importantly, when pre-selected STPH-transduced ES cells were applied, GCV treatment of the mice did not result in tumor formation (Figure 6B): only one out of six mice revealed the formation of a small (~0.3 cm) piece of muscle tissue that exhibited no other germ layer tissue (Table 1 and Figure 6C lower). These results clearly demonstrate that lentiviral mediated TK expression and GCV treatment can be used to eliminate even a large number of undifferentiated pluripotent cells *in vivo*.

**Table 1:** Summary of teratoma growth resulting from ES cells injections into hind limbs of SCID/beige mice*.

**Cell line^a^**	**Hygromycin selection^b^**	**GCV Treatment^c^**	**n**	**Teratoma > 1 cm**	**Teratoma < 1 cm**	**No teratoma**
**ES w/o virus**	-	-	9	8/9	1/9	-
**ES w/o virus**	-	+	11	11/11	-	-
**ES + STPH (L)**	+	-	4	4/4	-	-
**ES + STPH (L)**	+	+	6	-	1^d^ /6	5/6
**ES + STPH (H)**	-	-	5	5/5	-	-
**ES + STPH (H)**	-	+	5	1/5	4/5	-

^*^ The table summarizes the ES cells injections into hind limbs of SCID/beige mice and number of teratoma detected 3 weeks later. Teratoma size (>1 cm or <1 cm) indicates maximum dimension.

^a^ ES cells were transduced with STPH (+ STPH) or were not transduced (w/o virus).

L and H indicate ES cells carrying low (1.5) or high (3.8) copy number per genome in average.

^b^ Hygromycin pre-selected ES cells were cultured with 300 ng/ml hygromycin for 9 days before ES cell injection.

^c^ Mice were administrated with (+) or without (-) 20 mg/kg/day GCV for 12 days. GCV-untreated mice were administrated with same volume of 0.9% (w/v) NaCl.

^d^ Teratoma’s diameter is less than 0.3 cm and exhibit only muscle tissue (see also Figure 6C lower).

In addition, we also tested, high copy number STPH-transduced ES cells without pre-selection *in vivo*. Compared to the control group, receiving untransduced cells, a significant reduction in teratoma weight (around 65%) was observed for high copy number STPH-transduced ES cells without pre-selection when treated with GCV (Figure 6D). Furthermore, four out of five mice showed formation of only small teratomas upon GCV treatment (Table 1). In contrast, upon saline treatment all mice that were injected with STPH-transduced unselected ES cells, developed teratomas with maximum dimensions of more than 1 cm (Table 1). However, the complete absence of teratoma as observed for injection of pre-selected low copy number STPH-transduced ES cells was not achieved when using ES cells with high copy number LV TK (see also Figure 6B). These results confirmed our *in vitro* findings (see also Figure 5E and Supplementary Figure S4C). Thus, increasing the copy number of LVs does not per se result in a complete transduction of ES cell populations.

The data obtained suggested a promising future for the *in vivo* application of STPH-transduced ES cell lines with pre-selection as ES cells that have teratoma-forming potential seemed to be completely eliminated due to GCV application.

In further experiments, we started GCV treatment 7 days after ES cell injection (Figure S5). First, we analyzed tumor growth 7 days after ES cell injection of mice that were not treated with GCV (Figure S5A). In the absence of GCV, both untransduced as well as STPH-transduced ES cells gave rise to teratomas with no significant difference (teratomas size of approximately 4 mm). These results clearly showed the formation of teratomas within 7 days if no further treatment was applied. Hygromycin selection per se does not affect teratoma development of ES cells. Next, we compared teratoma formation of saline or GCV-treated mice that were injected with not transduced or STPH-transduced ES cells (Figure S5B). The treatment started 7 days after cell injection, when small tumors already had developed (see also Figure S5A). No significant differences in tumor sizes between injected untransduced and STPH-transduced and pre-selected cells upon GCV treatment could be observed showing that the TK/GCV system is not able to eliminate developed tumors. Our results underline the necessity of GCV application directly after ES cell injection to prevent tumor formation caused by pluripotent cells.

## Discussion

Pluripotent stem cells are characterized by their ability to self-renew and capability to differentiate into all cell types of the adult body. Due to these characteristics they are a promising cell source for regenerative medicine. However, former studies demonstrated the risk of teratoma formation in ES cell-based therapies [4] which remains a major safety obstacle that has to be solved before pluripotent cell-derived tissues can be applied in clinical trials. To avoid tumorigenesis of undifferentiated pluripotent cells different approaches have been developed. One method is the use of suicide genes such as the TK/GCV system to eliminate undifferentiated pluripotent cells [9,10,43]. Further possibilities rely on selection of residual pluripotent cells by application of specific surface markers and subsequent cell sorting [15] or on use of a specific antibody for elimination of pluripotent cells [13,14]. However, using surface markers to select residual pluripotent cells can only be applied *in vitro*. The TK/GCV system on the other hand, leads to elimination of undifferentiated pluripotent cells during the differentiation process *in vitro* as well as to removal of undifferentiated pluripotent stem cells *in vivo*. TK/GCV based therapies have already been used *in vivo* and are tested in clinical trials for e.g. the treatment of prostate cancer [44] or ovarian cancer [45].

To obtain stable transgene expression, the use of integrating LVs is a suitable tool for efficient gene transfer in a broad spectrum of stem cells including murine ES [22] and iPS cells [29] for both *in vitro* as well as *in vivo* applications [46]. In the present study, we used LVs carrying different promoters of pluripotency genes (Nanog, Oct, EOS-C3, EOS-S4) upstream of the TK cDNA in order to specifically eliminate pluripotent stem cells. In contrast, using ubiquitous promoters such as the phosphoglycerate kinase (PGK) promoter or the cytomegalovirus (CMV) promoter to drive TK expression would lead to elimination of all transduced cells and is therefore not useful [9].

Integrating viral vectors like LVs carry the risk of insertional mutagenesis [36,47]. Therefore, on the one hand low integration numbers in transduced pluripotent stem cells are desired. On the other hand, this increases the likelihood of untransduced cells that do not express TK and subsequently not all undifferentiated cells are eliminated upon GCV treatment. In the present study, we tried to find possible solutions for this dilemma by applying different approaches. In addition, motivation for this study was establishment of a protocol with ready-to-use LVs in combination with the TK/GCV system that could be applied to different pluripotent stem cell lines. This would facilitate a possible application in clinical trials.

In the first step, we applied TK expressing LVs to both ES and iPS cells to obtain infected cell populations that were achieved without further selection or clonal expansion. This resulted under undifferentiation conditions for both stem cell populations in elimination of cells after GCV treatment. We focused on ES cells that were transduced with certain amounts of LVs leading to a relatively low average copy number of approximately 1.5 per genome. Treatment of these NT- or OT-transduced mixed ES cell populations with GCV resulted in a survival of around 3 to 4% Oct-3/4-positive cells after differentiation. The presence of Oct-3/4-positive cells could be due to an insufficient TK expression level of OT- or NT-transduced ES cells or due to remaining untransduced ES cells. Analysis of different promoters to raise TK expression level revealed that EOS-S4 led to the highest TK abundance among the promoters tested. However, we could not observe higher killing efficiency of undifferentiated cells upon GCV treatment *in vitro*. Therefore, it is more likely that survival of undifferentiated cells is the consequence of untransduced cells that remain unaffected upon GCV treatment. To ensure that all ES cells contain the TK cDNA, we generated LV single ES cell clones by picking cell clones and further dispending the colonies. The clones were screened using Southern Blot analysis with a lentiviral specific probe and clones with a single integration were further investigated. Whereas almost no survival of undifferentiated cells was obtained for differentiated single integrant clones OT #4 and OT# 11, more than 1% of Oct-3/4 positive cells were still observed after GCV treatment of NT clones (NT #8 and NT #11). This incomplete elimination might be due to an epigenetic silencing of the transgene [29,48]. Another explanation for the presence of Oct-3/4 positive cells after GCV treatment and differentiation might be contaminations by a few untransduced ES cells (i.e. mixed cell clone). To avoid this, the single integrant clones can be further subcloned to achieve pure single integrant ES cell clones but this is more time consuming. Furthermore, in contrast to the generation of single clones, the usage of mixed ES cell populations is much simpler to handle since the additional process of clone selection and screening by Southern blot is not necessary. Irrespectively whether the TK vector is not functional (silencing) or not present (contamination with untransduced cells), a pre-selection strategy can be employed to obtain pure ES cell populations that express TK. Therefore, we incorporated hygromycin resistance gene as a pre-selection tool in the LVs (STPH vector) to select for transduced ES cells. And indeed, treatment of STPH-transduced ES cells with hygromycin and GCV abolished undifferentiated cells *in vitro*. Importantly, no teratoma formation in the *in vivo* studies could be observed when mice were injected with these pre-selected transduced ES cells and treated with GCV. This could be a promising application of LVs and the TK/GCV system in future clinical approaches. The GCV treatment has to be applied directly after ES cell injection to prevent tumor formation caused by pluripotent cells as it is not able to eliminate or stop the growth of established tumors. This in accordance with previously published results [49].

In addition, we tried to raise copy numbers of STPH-transduced ES cells to decline the untransduced cell population. Even with higher average copy numbers (approximately 3.8), several ES cells seemed to be not transduced because undifferentiated cells survived the GCV treatment. In conclusion, complete elimination was only achieved by hygromycin pre-selection.

## Conclusions

In summary, we successfully generated pluripotent stem cells expressing the TK gene after LV transduction. Our *in vitro* and *in vivo* data clearly demonstrated an efficient killing effect of undifferentiated pluripotent stem cells by GCV treatment by using low copy number STPH-transduced ES cell pre-selected with hygromycin. This in turn eliminated the risk of teratoma formation in the hind limbs of GCV treated mice *in vivo*. Moreover, our *in vitro* data also suggests interchanging of iPS cells for ES cells averting the controversial ethical application of human ES cells.

## Supporting Information

Figure S1 Immunostaining of dissociated EBs derived from mixed ES cell populations transduced with lentiviral NT or OT with low copy number. The ES cells transduced with NT or OT leading to 1.5 copy numbers per genome in average (as shown in Fig. 2B) were differentiated as EBs and treated with (+) or without (-) 20 µM GCV. After 14 days of differentiation dissociated EBs were immunostained with antibody against skeletal α-actinin (sarcomere) indicating the successful formation of cardiomyocytes (white); green fluorescence indicates eGFP expression driven by cardiac specific promoter, α-MHC.
(TIF)

Figure S2 Analysis of single LV-integrant NT or OT transduced ES cell clones *in vitro*. (**A**) and (**B**) ES cells were transduced with NT (A) or OT (B) and picked clones were analyzed by Southern Blot. Marked are the results of the single LV integrant ES cell clones. NT transduced ES cell clones are highlighted by white arrows. Unspecific band is indicated by asterisk (*). (**C**) Immunostaining of dissociated EBs of single integrant ES cell clones from (A) or (B) with (+) or without (-) GCV treatment with antibody against skeletal α-actinin (sarcomere) indicating the successful formation of cardiomyocytes (white); green fluorescence indicates eGFP expression driven by cardiac specific promoter α-MHC.
(TIF)

Figure S3 Analysis of STPH-transduced ES cells upon different time frames of pre-selection. ES cells were transduced with STPH (1.5 copy numbers per genome in average) or not transduced (WT) and treated with (+) or without (-) 20 µM GCV for 72 hours after pre-selection with Hygromycin (+Hygro) or without it (-Hygro) under undifferentiation conditions. (**A**) Relative cell survival of untransduced ES cells (WT). (**B**) Relative cell survival of STPH-transduced ES cells with 9 days Hygromycin pre-selection. (**C**) Relative cell survival of STPH-transduced ES cells with 6 days Hygromycin pre-selection followed by 3 days without Hygromycin treatment. (**D**) Relative cell survival of STPH-transduced ES cells with 9 days Hygromycin pre-selection followed by 3 days without Hygromycin treatment (n=3 in duplicates, respectively). Undifferentiated cells were manually counted using three different fields of view that were counted twice. Mean±SEM; ***P<0.001 compared to without GCV treatment, respectively, Student’s *t*-test).
(TIF)

Figure S4 Analysis of STPH-transduced ES cells with high copy number. (**A**) ES cells were transduced with different concentrations of LVs STPH leading to 3.8 copy number (high copy number) per genome in average or not transduced (WT) and treated with (+) or without (-) 20 µM GCV for 72 hours. Representative brightfield images are shown (n=3). (**B**) and (**C**) Relative cell survival of untransduced ES cells (WT) (B) or STPH-transduced ES cells (3.8 copy numbers per genome in average) without pre-selection (- Hygro) (C) with (+) or without (-) 20 µM GCV treatment (n=3, Mean±SEM; ***P<0.001 compared to without GCV treatment, respectively, Student’s *t*-test). Data is based on images representatively shown in (A), undifferentiated cells were manually counted using three different fields of view that were counted twice.
(TIF)

Figure S5 Analysis of injection of untransduced or STPH-transduced ES cells with delayed GCV treatment. 1 x 10^6^ ES cells (STPH-transduced with 1.5 copy numbers per genome in average and pre-selected with hygromycin for 9 days or not transduced (WT)) were injected s.c. into hind limbs of SCID/beige mice. (**A**) Relative weight of teratomas was analyzed 7 days after cell injection (n=3, Mean±SEM). (**B**) 7 days after cell injection mice were administrated i.p. with saline solution (0.9% (w/v) NaCl; -GCV) or with GCV (20 mg/kg/day, +GCV) for consecutive 12 days and relative weight of teratomas was finally analyzed (n=6, Mean±SEM).
(TIF)
