# Supplementary figures and images for: Lentiviral Vector Mediated Thymidine Kinase Expression in Pluripotent Stem Cells Enables Removal of Tumorigenic Cells
Source: PLoS One. 2013 Jul 30;8(7):e70543. doi: 10.1371/journal.pone.0070543 (PMC3728319; doi:10.1371/journal.pone.0070543)

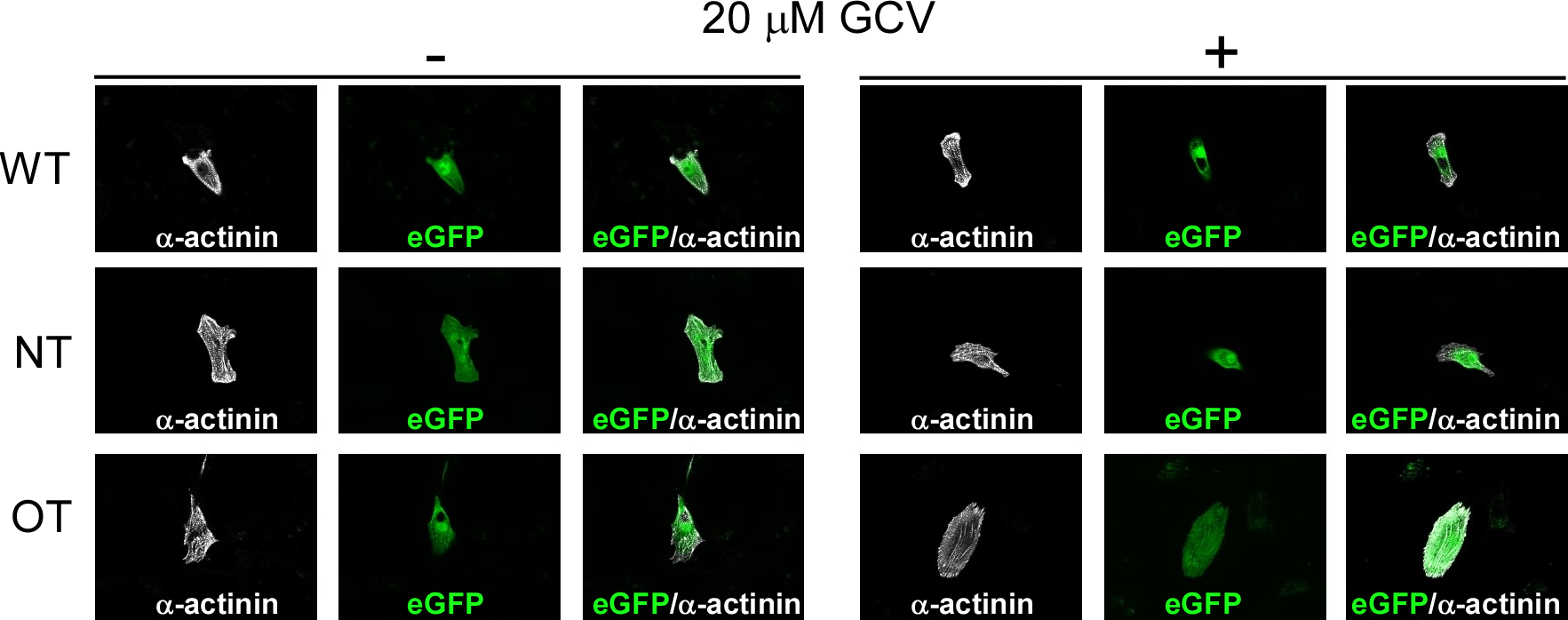

Supplement: Figure S1 — The ES cells transduced with NT or OT leading to 1.5 copy numbers per genome in average (as shown in Fig. 2B) were differentiated as EBs and treated with (+) or without (-) 20 µM GCV. After 14 days of differentiation dissociated EBs were immunostained with antibody against skeletal α-actinin (sarcomere) indicating the successful formation of cardiomyocytes (white); green fluorescence indicates eGFP expression driven by cardiac specific promoter, α-MHC. (TIF) [file pone.0070543.s001.tif]

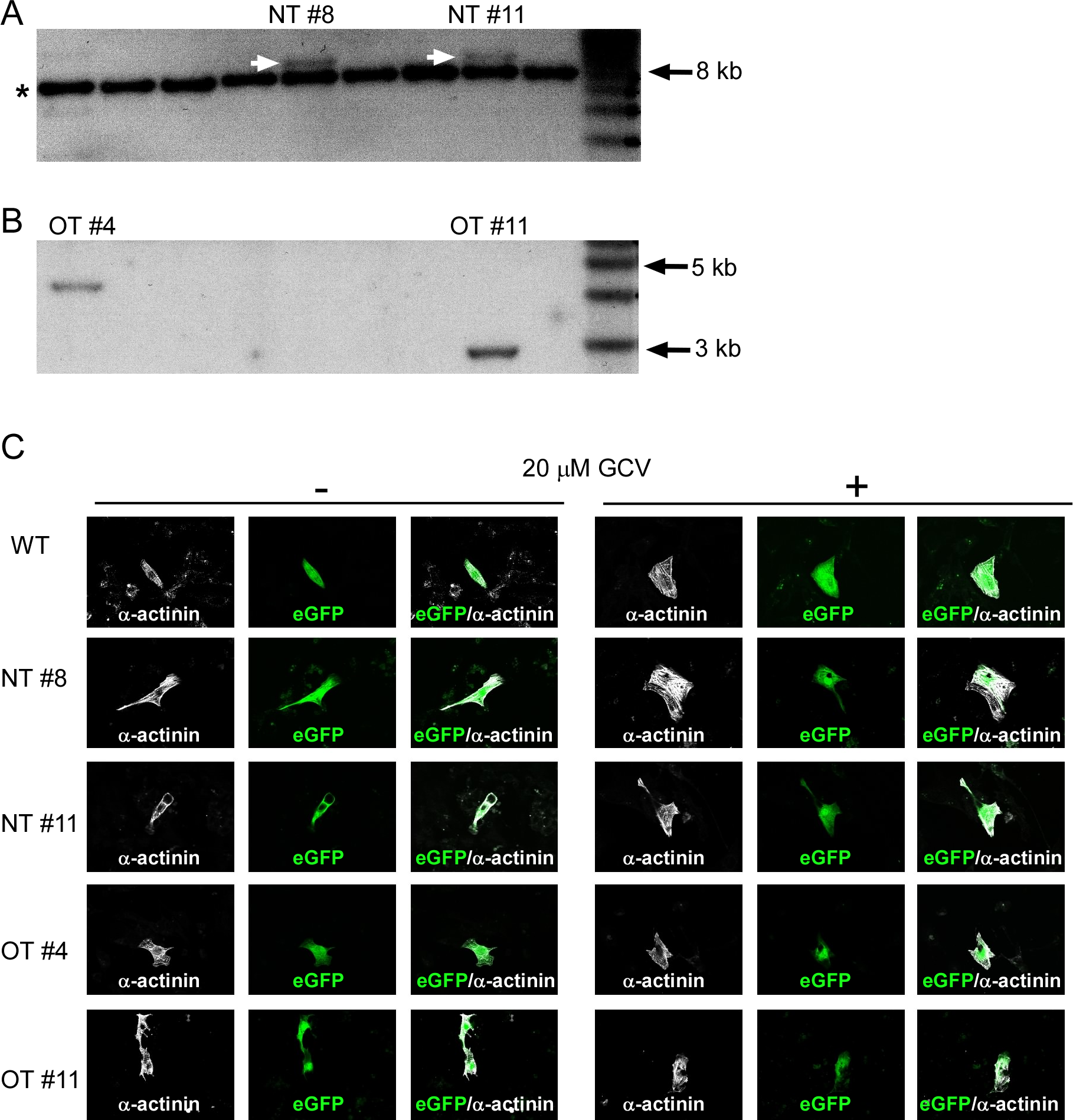

Supplement: Figure S2 — (A) and (B) ES cells were transduced with NT (A) or OT (B) and picked clones were analyzed by Southern Blot. Marked are the results of the single LV integrant ES cell clones. NT transduced ES cell clones are highlighted by white arrows. Unspecific band is indicated by asterisk (*). (C) Immunostaining of dissociated EBs of single integrant ES cell clones from (A) or (B) with (+) or without (-) GCV treatment with antibody against skeletal α-actinin (sarcomere) indicating the successful formation of cardiomyocytes (white); green fluorescence indicates eGFP expression driven by cardiac specific promoter α-MHC. (TIF) [file pone.0070543.s002.tif]

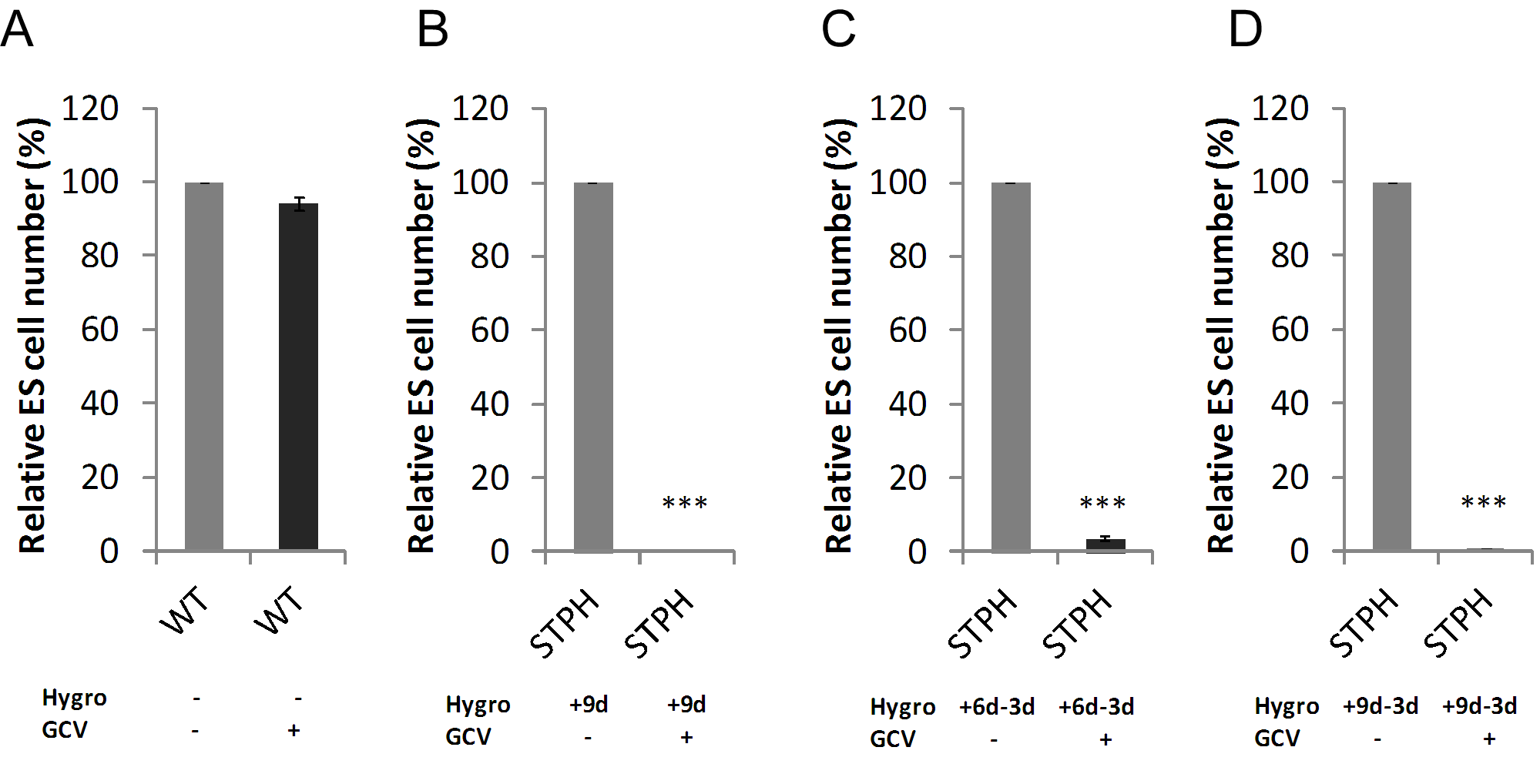

Supplement: Figure S3 — ES cells were transduced with STPH (1.5 copy numbers per genome in average) or not transduced (WT) and treated with (+) or without (-) 20 µM GCV for 72 hours after pre-selection with Hygromycin (+Hygro) or without it (-Hygro) under undifferentiation conditions. (A) Relative cell survival of untransduced ES cells (WT). (B) Relative cell survival of STPH-transduced ES cells with 9 days Hygromycin pre-selection. (C) Relative cell survival of STPH-transduced ES cells with 6 days Hygromycin pre-selection followed by 3 days without Hygromycin treatment. (D) Relative cell survival of STPH-transduced ES cells with 9 days Hygromycin pre-selection followed by 3 days without Hygromycin treatment (n=3 in duplicates, respectively). Undifferentiated cells were manually counted using three different fields of view that were counted twice. Mean±SEM; ***P<0.001 compared to without GCV treatment, respectively, Student’s t-test). (TIF) [file pone.0070543.s003.tif]

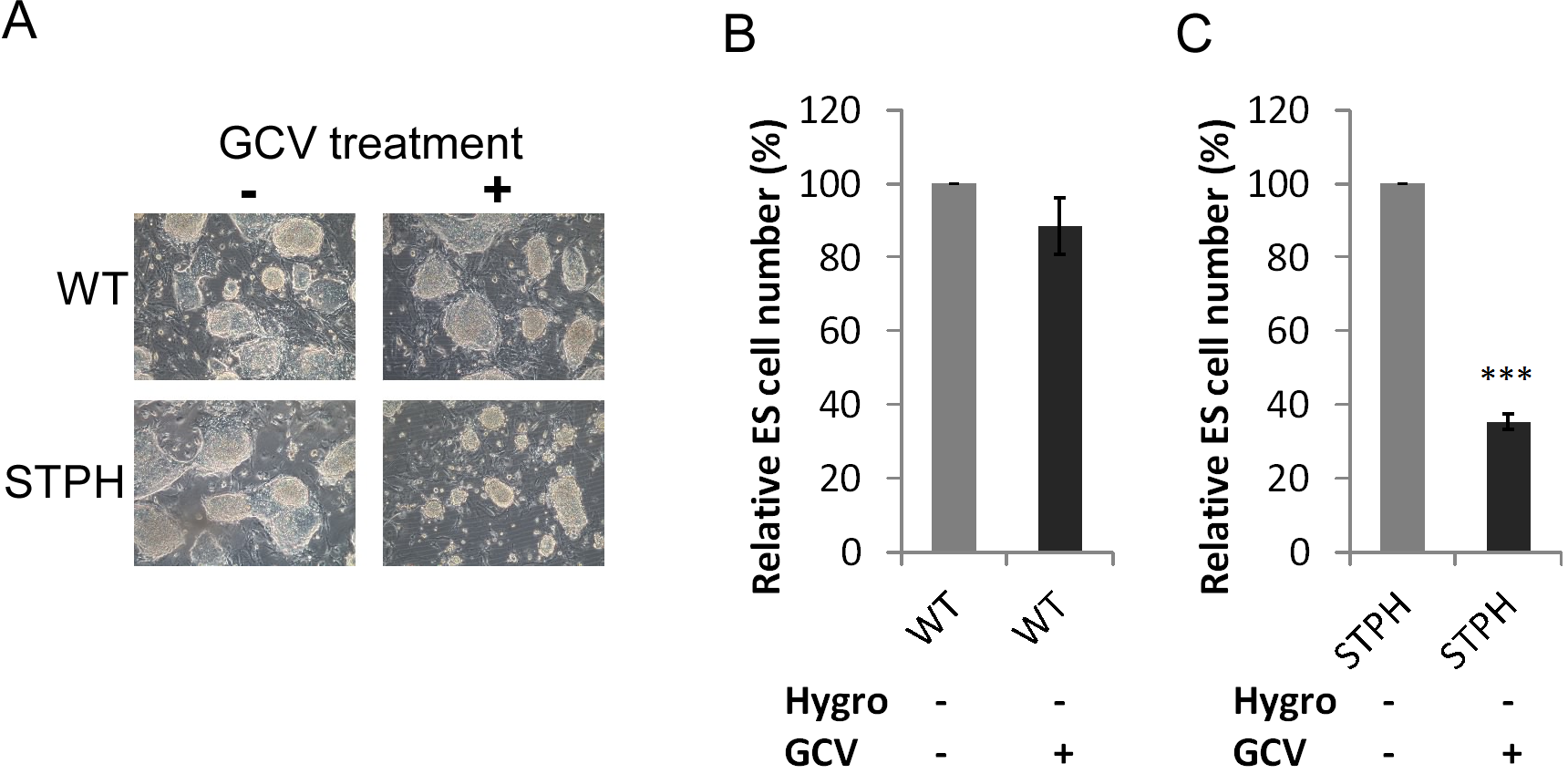

Supplement: Figure S4 — (A) ES cells were transduced with different concentrations of LVs STPH leading to 3.8 copy number (high copy number) per genome in average or not transduced (WT) and treated with (+) or without (-) 20 µM GCV for 72 hours. Representative brightfield images are shown (n=3). (B) and (C) Relative cell survival of untransduced ES cells (WT) (B) or STPH-transduced ES cells (3.8 copy numbers per genome in average) without pre-selection (- Hygro) (C) with (+) or without (-) 20 µM GCV treatment (n=3, Mean±SEM; ***P<0.001 compared to without GCV treatment, respectively, Student’s t-test). Data is based on images representatively shown in (A), undifferentiated cells were manually counted using three different fields of view that were counted twice. (TIF) [file pone.0070543.s004.tif]

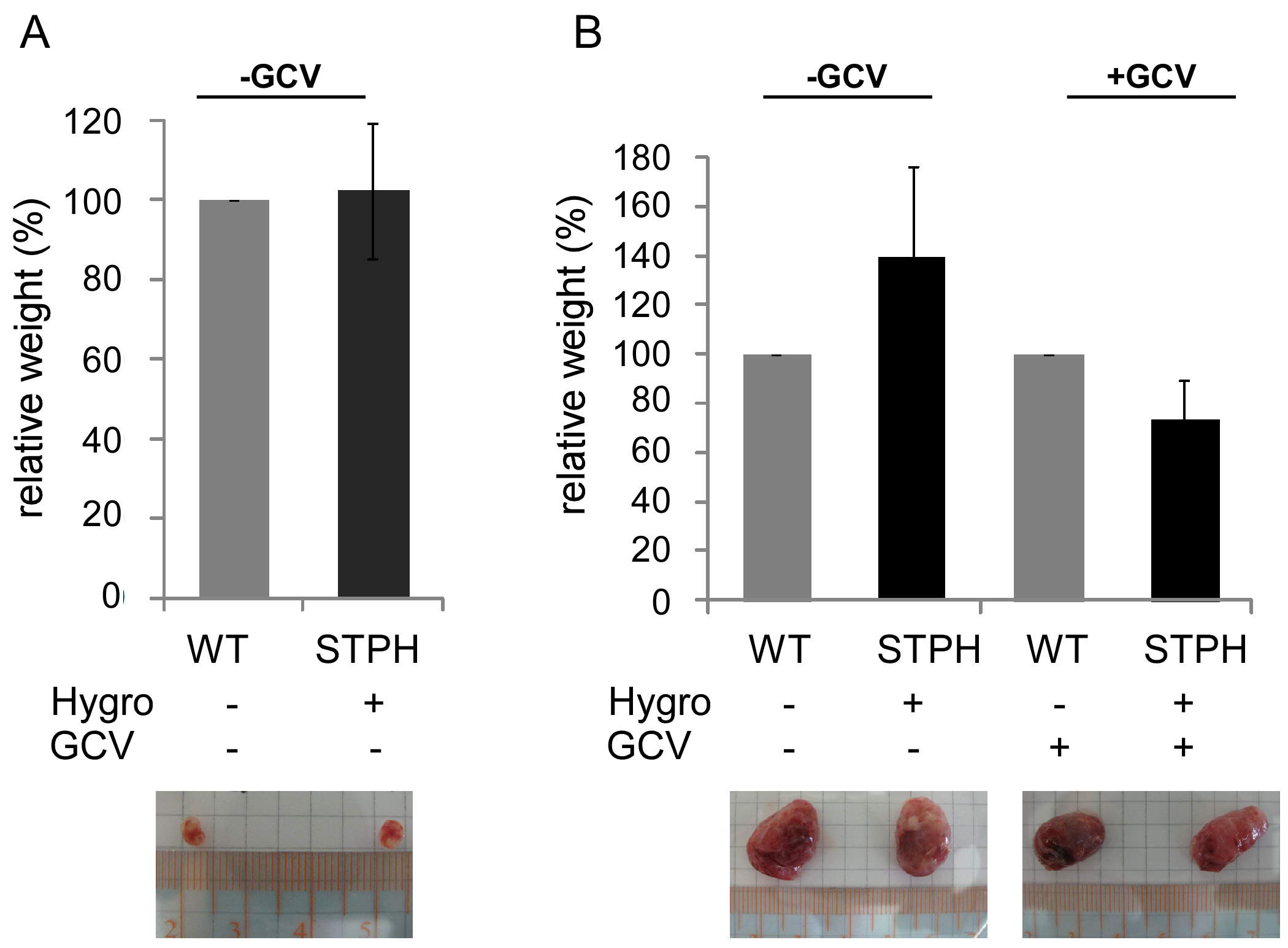

Supplement: Figure S5 — 1 x 10^6^ ES cells (STPH-transduced with 1.5 copy numbers per genome in average and pre-selected with hygromycin for 9 days or not transduced (WT)) were injected s.c. into hind limbs of SCID/beige mice. (A) Relative weight of teratomas was analyzed 7 days after cell injection (n=3, Mean±SEM). (B) 7 days after cell injection mice were administrated i.p. with saline solution (0.9% (w/v) NaCl; -GCV) or with GCV (20 mg/kg/day, +GCV) for consecutive 12 days and relative weight of teratomas was finally analyzed (n=6, Mean±SEM). (TIF) [file pone.0070543.s005.tif]
